# Copper-Zinc-Tin-Sulfur Thin Film Using Spin-Coating Technology

**DOI:** 10.3390/ma9070526

**Published:** 2016-06-29

**Authors:** Min-Yen Yeh, Po-Hsun Lei, Shao-Hsein Lin, Chyi-Da Yang

**Affiliations:** 1 Department of Microelectronic Engineering, National Kaohsiung Marine University, Kaohsiung 811, Taiwan; minyen@mail.nkmu.edu.tw (M.-Y.Y.); cdyang@webmail.nkmu.edu.tw (C.-D.Y.); 2 Institute of Electro-Optical and Materials Science, National Formosa University, 64 Wen-Hwa Rd, Hu-Wei, Yun-Lin 632, Taiwan; id7870.sl@gmail.com

**Keywords:** CZTS thin film, solar cell, optical band gap, conversion efficiency

## Abstract

Cu_2_ZnSnS_4_ (CZTS) thin films were deposited on glass substrates by using spin-coating and an annealing process, which can improve the crystallinity and morphology of the thin films. The grain size, optical gap, and atomic contents of copper (Cu), zinc (Zn), tin (Sn), and sulfur (S) in a CZTS thin film absorber relate to the concentrations of aqueous precursor solutions containing copper chloride (CuCl_2_), zinc chloride (ZnCl_2_), tin chloride (SnCl_2_), and thiourea (SC(NH_2_)_2_), whereas the electrical properties of CZTS thin films depend on the annealing temperature and the atomic content ratios of Cu/(Zn + Sn) and Zn/Sn. All of the CZTS films were characterized using X-ray diffraction (XRD), scanning electron microscopy (SEM), energy-dispersive X-ray spectroscopy (EDXS), Raman spectroscopy, and Hall measurements. Furthermore, CZTS thin film was deposited on an n-type silicon substrate by using spin-coating to form an Mo/p-CZTS/n-Si/Al heterostructured solar cell. The p-CZTS/n-Si heterostructured solar cell showed a conversion efficiency of 1.13% with *V*_oc_ = 520 mV, *J*_sc_ = 3.28 mA/cm^2^, and fill-factor (FF) = 66%.

## 1. Introduction

Semiconductor solar cells incorporating thin-film absorbers are an attractive option for replacing classical silicon-based solar cells. In recent years, several thin-film absorbers such as III–V compound material (GaInP/GaAs) [1,2,3], I–III–VI compound material (Cu(In, Ga)Se_2_, CIGS) [4,5,6], and I–II–VI compound material (Cu_2_ZnSnS_4_, CZTS) [7,8,9] have been investigated for semiconductor thin-film solar cells. Among these studied materials, direct band gap chalcopyrite-structured CIGS and kesterite-structured CZTS are potential absorber materials for the next generation of thin-film solar cells because of their large optical absorption coefficients over the visual light range. A record conversion efficiency of 20.3% was reported for a CIGS thin-film solar cell [4]. However, use of toxic Se and scarce or expensive elements, such as gallium (Ga) and indium (In), may limit the production development of CIGS thin-film solar cells. Researchers strongly desire to identify a nontoxic inexpensive thin-film absorber with high conversion efficiency to replace CIGS. CZTS has been considered as an alternative to CIGS because of its majority carrier type (p-type), direct band gap of 1.4–1.5 eV, and large optical absorption coefficient (>10^4^ cm^−1^) over the visible light range [10]. In addition, CZTS thin films are composed of elements that are nontoxic, abundant, and inexpensive, namely copper, zinc, tin, and sulfur.

CZTS thin films are generally fabricated using vacuum-based techniques such as thermal evaporation [11], sputtering [12], and pulsed laser deposition [13]. These methods can deposit high-quality CZTS thin films on molybdenum (Mo)-coated glass substrates. However, these techniques require complicated equipment to maintain a vacuum and high process temperatures. In addition to high conversion efficiency, film content and composition as well as large-scale rather than small-area cell structural uniformity are challenges in developing CZTS thin-film solar cells for industrial use. Many nonvacuum methods of reducing production costs and satisfying the large-area requirements have been studied, including electrodeposition [14], spray pyrolysis techniques [15], sol-gel processing [16], spin-coating methods [17], and chemical bath methods [18]. Among these, the spin-coating method has the advantages of simple construction, low cost, ecological safety, and the large-scale deposition of different semiconductor thin films. However, few studies have reported the preparation of spin-coated CZTS thin films without toxic precursors such as hydrazine [19] or ethanolamine (MEA) [17]. Researchers do not completely and clearly understand the relationships of the deposition parameters to the atomic contents of Cu, Zn, Sn, and S, surface morphology, and electrical properties of spin-coated CZTS thin films. 

In this study, we developed spin-coating Cu-poor and Zn-rich CZTS thin films with optimal atomic contents of Cu, Zn, Sn, and S onto glass and silicon (Si) substrates. The atomic contents of Cu, Zn, Sn, and S, crystalline morphology, and surface morphology of these CZTS thin films were characterized using energy-dispersive X-ray spectroscopy (EDX), X-ray diffraction (XRD) patterns, field-emission scanning electron microscopy (FE-SEM) images, and Raman measurements. The optical band gap and electrical properties were measured using absorption spectra and Hall measurements. Finally, an optimal CZTS thin was spun on an n-type Si substrate to form a Mo/p-CZTS/n-Si/Al solar cell.

## 2. Results and Discussion

**Figure 1:**
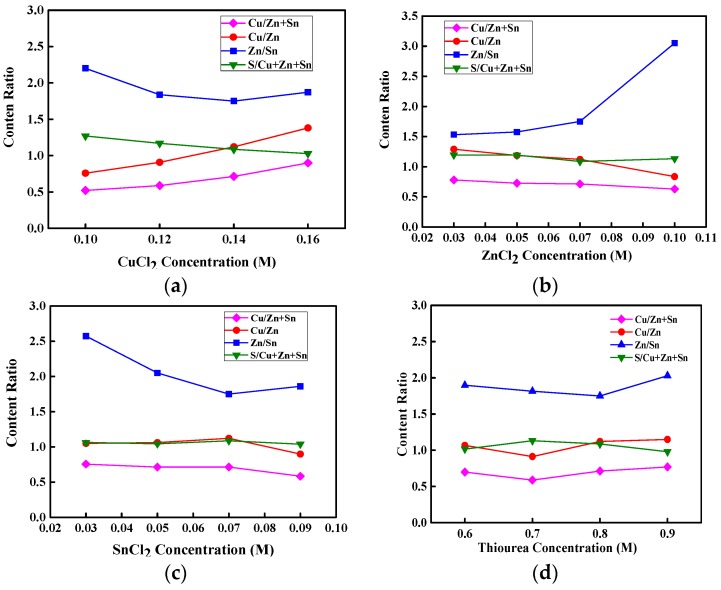
Atomic content ratios of Cu/(Zn + Sn), Cu/Zn, Zn/Sn, and S/(Cu + Zn + Sn) for copper-zinc-tin-sulfur (CZTS) thin films with (**a**) CuCl_2_; (**b**) ZnCl_2_; (**c**) SnCl_2_; and (**d**) thiourea concentrations, respectively.

The crystallinity, morphology, electrical properties, and band gap energy of CZTS thin films show a strong dependence on the stoichiometry of Cu, Zn, Sn, and S in those CZTS thin films [9,16,20,21,22]. Well-controlled atomic contents of Cu, Zn, Sn, and S in CZTS thin films has become a crucial issue for fabricating high-performance kesterite CZTS solar cells. To determine the optimal atomic contents of Cu, Zn, Sn, and S in CZTS thin films, the chemical concentrations of CuCl_2_, ZnCl_2_, SnCl_2_, and thiourea aqueous precursor solution should be analyzed. Figure 1a–d show the atomic content ratios of Cu/(Zn + Sn), Cu/Zn, Zn/Sn, and S/(Cu + Zn + Sn) for CZTS thin films as functions of the concentrations of CuCl_2_, ZnCl_2_, SnCl_2_, and thiourea aqueous precursor solution, respectively, for an annealing temperature of 350 °C. As shown in Figure 1a–c, the atomic contents of Cu, Zn, and Sn increase with raising the concentration of CuCl_2_, ZnCl_2_, and SnCl_2_ aqueous precursor solution, respectively, implying that the atomic contents of Cu, Zn, Sn, and S in CZTS thin films can be modulated by changing the concentrations of aqueous precursor solution. Cu-rich CZTS thin films can facilitate the formation of passivated defect clusters such as Cu_Zn_ + Sn_Zn_ and 2Cu_Zn_ + Sn_Zn_. The former produces a deep donor level in the band gap of CZTS, whereas the latter can significantly reduce the band gap of CZTS [20]. However, a high Zn/Sn ratio or high Zn atomic content is necessary to form the Zn_Sn_ acceptors and reduce the Sn_Zn_ donors to increase the conductivity of the CZTS thin film [23]. However, high Zn atomic content, which results in a low Cu/Zn ratio, can bring about a p/n conduction type [22]. To avoid defect clusters and increase the conductivity of p-type CZTS thin films, the Cu/(Zn + Sn) atomic content ratio should be less than 1, the Zn/Sn atomic content ratio should be more than 1, and the Cu/Zn atomic content ratio should not be too low. In Figure 1a, the atomic content ratio of Cu/(Zn + Sn) is lower than 1 for the CuCl_2_ concentration below 0.14 M and approaches 1 at the CuCl_2_ concentration of 0.16 M. When the ZnCl_2_, SnCl_2_, and thiourea concentrations increase with a CuCl_2_ concentration of 0.14 M, the Cu/(Zn + Sn) atomic content ratio remains below 1, as seen in Figure 1b–d. These results indicate that Cu-poor and Zn-rich CZTS thin films can be produced from properly controlled concentrations of aqueous precursor solutions. The atomic content ratio of S/Cu + Zn + Sn in CZTS thin films can approach 1 for the concentrations of CuCl_2_, ZnCl_2_, and SnCl_2_, as shown in Figure 1a–c, respectively. In addition, in Figure 1d, the atomic content of S increases slightly as the thiourea concentration rises from 0.6 to 0.8 M, and saturates at a thiourea concentration above 0.8 M, possibly because of the solubility of S in the CZT thin film. 

**Figure 2:**
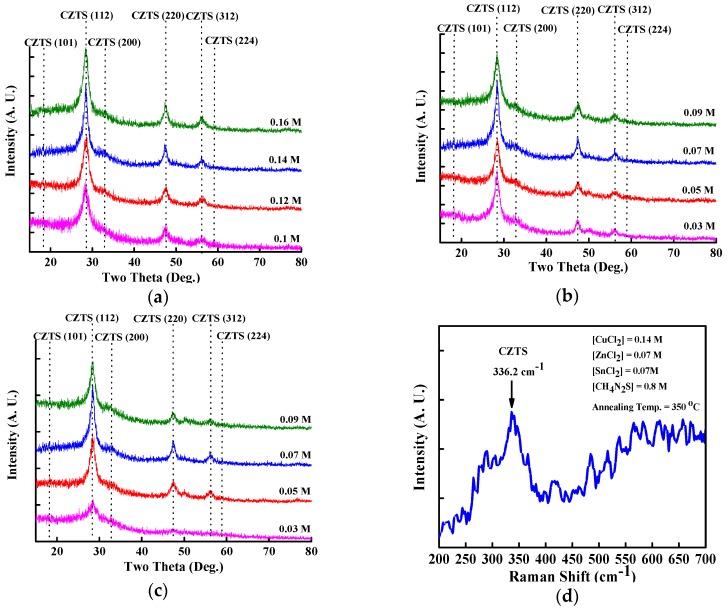
X-ray diffraction (XRD) patterns for CZTS thin film with varied concentration of (**a**) CuCl_2_; (**b**) ZnCl_2_; and (**c**) SnCl_2_ aqueous precursor solution under an annealing temperature of 350 °C. The Raman spectrum for a CZTS thin film with CuCl_2_, ZnCl_2_, SnCl_2_, and thiourea aqueous precursor solution concentration of 0.14, 0.07, 0.07, and 0.8 M, respectively, after annealing at 350 °C is shown in (**d**).

Figure 2a–c represent the XRD patterns for films with various concentrations of CuCl_2_, ZnCl_2_, and SnCl_2_ aqueous precursor solutions that were annealed at a temperature of 350 °C. The XRD peaks were indexed using JADE software (Jade 5.0, Christchurch, New Zealand) as the CZTS kesterite structure; and they closely matched the standard data (PDF#26-5075). All of the spin-coated CZTS thin films exhibited a polycrystalline kesterite crystal structure with six broad peaks along the (101), (112), (200), (220), (312) and (224) planes as shown in Figure 2a–c. The secondary phases including Cu_2_SnS_3_, CuS, SnS and ZnS would form during growth of CZTS thin films. They will degrade the performance of CZTS solar cells. It is important to avoid the formation of secondary phases in CZTS thin films. Among these secondary phases, tetragonal-structured Cu_2_SnS_3_ (PDF#33-0501) and cubic-structured ZnS (PDF#05-0566) show the similar XRD patterns to kesterite-structured CZTS between diffraction angles of 20 and 80 degrees because of the same lattice constants [15]. As a result, the measured XRD patterns of CZTS thin film might be related to either CZTS or Cu_2_SnS_3_ or ZnS pahse. The weak peak at 18.2° attributed to (101) plane indicates a kesterite-structured CZTS thin film because it is not found in the XRD patterns of tetragonal-structured Cu_2_SnS_3_ and cubic-structured ZnS. Consequently, a CZTS thin film without secondary phase, which leads to a degraded performance of CZTS solar cells, can be obtained using properly controlled concentrations of aqueous precursor solutions and a proper annealing process. To further confirm the XRD characteristics of this spin-coated CZTS thin film, which differ from similar XRD patterns such as those of ZnS and Cu_2_SnS_3_, Raman spectroscopy was performed. Figure 2e shows the Raman spectrum for a spin-coated CZTS thin film prepared from 0.14 M CuCl_2_, 0.07 M ZnCl_2_, 0.07 M SnCl_2_, and 0.8 M thiourea aqueous precursor solutions and annealed at 350 °C. The characteristic CZTS peak in Figure 2e is at 336.2 cm^−1^, which is different from the Raman peaks of Cu_2–*x*_S at 475 cm^−1^, ZnS at 273 and 351 cm^−1^, Cu_2_SnS_3_ at 290 and 352 cm^−1^, and of Sn_2_S_3_ at 234 and 307 cm^−1^, indicating a spin-coated CZTS thin film deposited on glass substrate [24,25].

**Figure 3:**
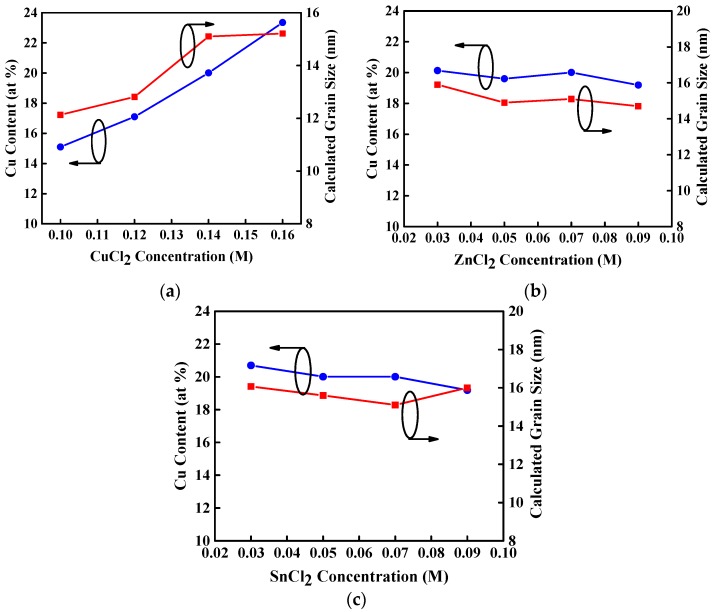
Full width at half maximum (FWHM) of (112) peak and calculated grain size for CZTS thin films with varied concentration of (**a**) CuCl_2_; (**b**) ZnCl_2_; and (**c**) SnCl_2_ aqueous precursor solution under an annealing temperature of 350 °C.

The mean crystallite grain size (D) of CZTS thin film can be estimated by the Scherrer formula [26]:

(1) $$D=c\frac{0.9\lambda }{\beta \cos\theta }$$

where β, λ, and θ_B_ are the line width at full width at half the maximum of the film diffraction peak at 2θ, X-ray wavelength (0.15406 nm), and Bragg diffraction angle, respectively. Figure 3a–c show the full width at half maximum (FWHM) of (112) peak and calculated mean crystallite grain size of CZTS thin films as a function of the varied concentration of CuCl_2_, ZnCl_2_, and SnCl_2_ aqueous precursor solutions that were annealed at a temperature of 350 °C. The grain size enlarges with increasing CuCl_2_ concentration, as shown in Figure 3a because of the increase of Cu atomic content in the CZTS thin films [27,28]. This increase in the grain size might be due to agglomeration of grains. In addition, the grain size of CZTS thin films represents a slight change as ZnCl_2_, and SnCl_2_ concentrations increase or decrease while CuCl_2_ concentration is maintained at 0.14 M, as shown in Figure 3b,c. This is attributed to the fairly constant Cu atomic content in these CZTS thin films. Consequently, the grain size of CZTS thin films with the same annealing temperature is determined by the Cu atomic content.

**Figure 4:**
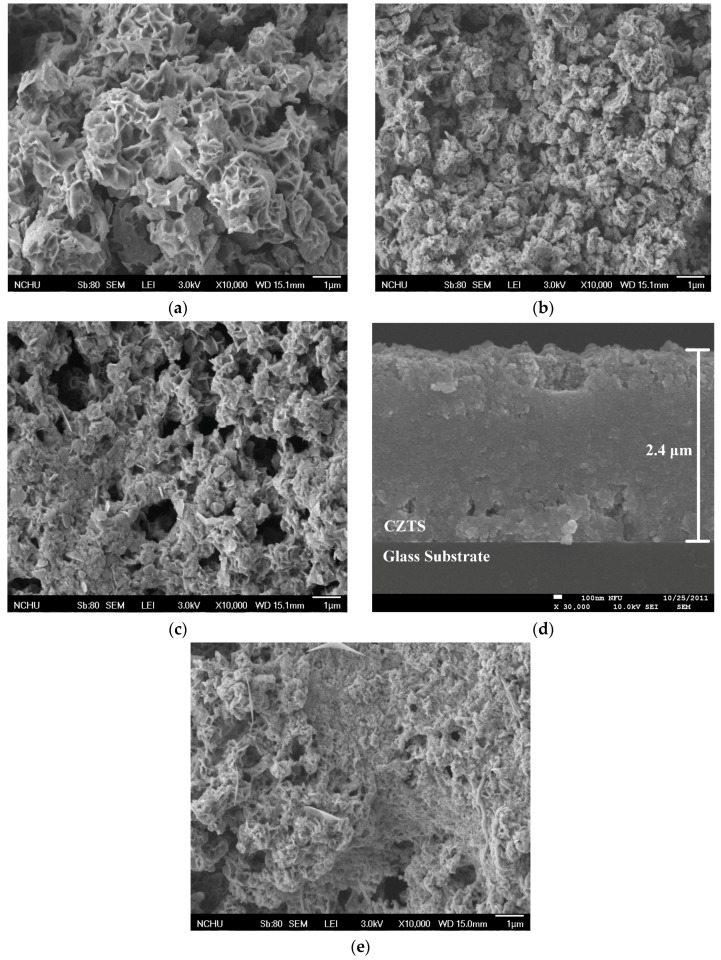
Plane-view field-emission scanning electron microscopy (FE–SEM) images of CZTS thin films deposited with ZnCl_2_, SnCl_2_, and thiourea concentrations of 0.07, 0.07, and 0.8 M, respectively, and CuCl_2_ concentrations of (**a**) 0.1 M; (**b**) 0.14 M; and (**c**) 0.16 M, respectively. The cross section image of a CZTS thin film with CuCl_2_, ZnCl_2_, SnCl_2_, and thiourea concentrations of 0.14, 0.07, 0.07, and 0.8 M is represented in (**d**). A CZTS thin film fabricated with ZnCl_2_, SnCl_2_, CuCl_2_, and thiourea concentrations of 0.1, 0.07, 0.14, and 0.8 M, respectively, is shown in (**e**).

The conversion efficiency of a CZTS thin-film solar cell is strongly associated with the grain size because an absorber layer with a large grain size maximizes the minority carrier diffusion length and the inherent potential of a polycrystalline solar cell [16,24]. Figure 4 shows the FE–SEM images of the CZTS thin film. Figure 4a–c show plane views of CZTS thin films with CuCl_2_ concentrations of 0.1, 0.14, and 0.16 M with concentrations of ZnCl_2_, SnCl_2_, and thiourea at 0.07, 0.07, and 0.8 M, respectively. The thickness of all the CZTS thin films was approximately 2.4 μm, and Figure 4d represents the FE-SEM cross section image of CZTS thin film with concentrations of CuCl_2_, ZnCl_2_, SnCl_2_, and thiourea at 0.14, 0.07, 0.07, and 0.8 M. As shown in Figure 4a–c, large numbers of voids were observed in the Cu-rich CZTS thin films. Voids of Cu-rich CZTS absorbers in thin-film solar cells cause low conversion efficiencies for photovoltaic applications [16,29]. Well-controlled Cu atomic content is important to obtain a large grain size and less number of voids in CZTS thin films. Figure 4e shows the plane view of a CZTS thin film with a high ZnCl_2_ concentration of 0.1 M and CuCl_2_, SnCl_2_, and thiourea concentrations of 0.14, 0.07, and 0.8 M. The surface of Zn-rich CZTS thin film is smoother than that of Zn-poor CZTS thin films because of the large columnar grains at the bottom [30].

**Figure 5:**
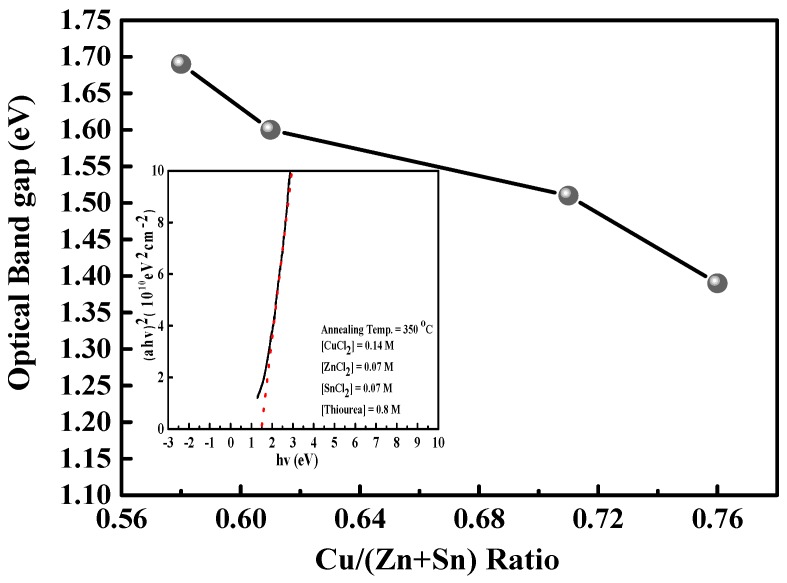
Optical band gap as a function of the Cu/(Zn + Sn) ratio, and the inset shows the plot of (αhν)^2^ against the photon energy of a Cu-poor and Zn-rich (Cu/Zn = 1.12) CZTS thin film.

Figure 5 shows the graph of optical band gap energy as a function of the Cu/(Zn + Sn) atomic content ratio. The optical band gaps of these CZTS thin films range from 1.39 to 1.69, whereas the Cu/(Zn + Sn) atomic content ratios range from 0.58 to 0.76. This shift is attributed to the change in the p-d hybridization between Cu d-levels and S p-levels [16]. In addition, the relation of the extended optical band gap to the decrease in grain size is attributed to the fact that the band gap energy of a material is dependent on the particle size of that material according to the quantum size effect [15]. As shown in Figure 3a–c, the grain size of a Cu-poor CZTS thin film is smaller than that of a Cu-rich CZTS thin film, resulting in a wide optical band gap. The inset of Figure 5 shows a plot of (αhν)^2^ against the photon energy of a Cu-poor and Zn-rich (Cu/Zn = 1.12) CZTS thin film where α is the absorption coefficient and hν is the photon energy. The optical band gap energy is estimated by extrapolating the linear region in the (αhν)^2^ plot and determining the intercept with the photon energy axis. The optical band gap for an optimal CZTS absorber is approximately 1.5 eV. 

**Figure 6:**
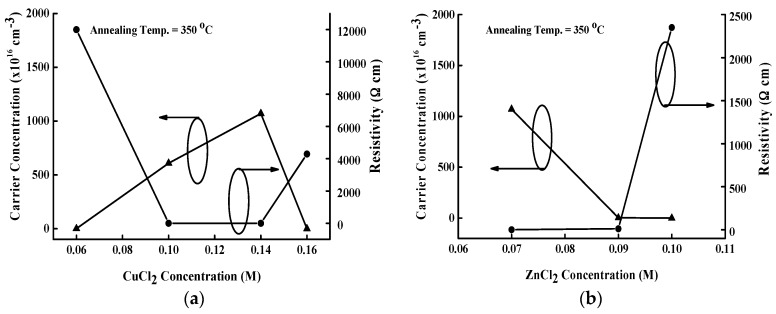
Carrier concentration and resistivity of the CZTS thin film as a function of the (**a**) CuCl_2_; and (**b**) ZnCl_2_ concentrations.

Figure 6a,b show the carrier concentration and resistivity of CZTS thin films as a function of CuCl_2_ and ZnCl_2_ concentration, respectively. The carrier concentration shown in Figure 6a increases with CuCl_2_ concentration. The formation of a Cu_Zn_ + Sn_Zn_ deep level depends on the Cu/(Zn + Sn) and Zn/Sn atomic content ratio, and an optimal condition occurs at Cu/(Zn + Sn) ≈ 0.8 and Zn/Sn ≈ 1.2 [20]. As the CuCl_2_ concentration increases from 0.1 to 0.14 M, the Cu/(Zn + Sn) atomic content ratio approaches the optimal value because of the suppression of the formation of a Cu_Zn_ + Sn_Zn_ deep donor level in the CZTS band gap, leading to a significant increase in hole concentration. Nevertheless, the Cu/(Zn + Sn) atomic content ratio increases with CuCl_2_ concentration to 1.6 M, causing the generation of a Cu_Zn_ + Sn_Zn_ deep donor defect and a reduction of the Cu_Zn_ antisite acceptor defects, resulting in a decrease of carrier concentration. Under the optimal Cu/(Zn + Sn) atomic content ratio of 0.714, the CZTS thin film has a high carrier concentration of 1.07 × 10^19^ cm^−3^ and a resistivity of 1.19 Ωcm. The atomic content ratio of Zn/Sn is also a crucial factor determining the electrical properties of a CZTS thin film. The Zn_Sn_ antisite defect with an inherent p-type conduction type in a CZTS thin film can easily form under a high Zn/Sn atomic content ratio. However, further increases in Zn/Sn atomic content ratio would generate a large population of intrinsic defects, causing a detrimental influence relative to a CZTS thin film with a atomic content ratio of Cu/(Zn + Sn) = 1 and Zn/Sn = 1. In Figure 6b, the hole concentration decreases as the ZnCl_2_ concentration rises from 0.7 to 0.9 M because of the population of donor defect clusters [20]. Further increases in ZnCl_2_ concentration would move the Cu/(Zn + Sn) atomic content ratio away from 0.8 (see Figure 1b), leading to a translation of conduction types.

The sulfer vacancy (V_S_) is also a dominant donor-like defect in a CZTS thin film because of its low formation energy. The conduction type would change from p-type to n-type and the hole concentration might decrease in an S-poor CZTS thin film due to a high concentration of V_S_. High S content or S-rich in CZTS thin film is crucial to reduce the concentration of V_S_. Kosyak et al. [31] have calculated the concentration of Zn_Sn_ antisite and V_S_ in CZTS thin films with different Cu, Zn, and Sn content, and represented that the concentration of acceptor-like Zn_Sn_ antisite is higher than that of donor-like V_S_ in a Zn-rich and Sn-poor CZTS thin film. Based on this result, we measure the electrical properties of CZTS thin films with various thiourea concentration under optimal Cu/(Zn + Sn) atomic content ratio of 0.714. Table 1 lists the measured results of S/(Cu + Zn + Sn) atomic content ratio and electrical characteristics of CZTS thin films depending on various thiourea concentration. The S/(Cu + Zn + Sn) atomic content ratio increases with rising thiourea concentration, implying that the a high S content or S-rich CZTS thin film can be obtained by changing the concentration thiourea aqueous solution. As thiourea concentration rises from 0.6 to 0.8 M, the hole concentration and conductivity of the p-type CZTS thin film increase because of reduction of V_S_ concentration. However, further increase in thiourea concentration would reduce the hole concentration and conductivity possible due to the formation of ZnS [32].

Table 2 shows the relation between annealing temperature and conduction type, carrier concentration, mobility, and resistivity of CZTS thin films with concentrations of CuCl_2_, ZnCl_2_, SnCl_2_, and thiourea aqueous precursor solution at 0.14, 0.07, 0.07, and 0.8 M, respectively. The hole concentration for annealing temperatures of 300 °C, 350 °C, 400 °C, and 450 °C are 2.02 × 10^14^, 1.07 × 10^19^, 2.80 × 10^15^, and 1.24 × 10^16^ cm^−3^. The varied carrier concentrations of CZTS thin films with increasing annealing temperatures is attributed to the changes of Cu/(Zn + Sn) and Zn/Sn content ratios, which cause the formation of Cu_Zn_ + Sn_Zn_ deep donor defects by reducing the Cu_Zn_ antisite acceptor defects [20,25]. The mobility of CZTS thin films shows a counterclockwise dependence on the carrier concentration because of the impurity scattering. In conclusion, with an annealing temperature of 350 °C, CZTS thin films have a stable p-type conduction type with a high hole concentration of 1.07 × 10^19^ cm^−3^ and a low resistivity of 1.19 Ωcm. To realize the optimal CZTS absorber for photovoltaic solar cell applications, a CZTS thin film was spin-coated onto an Si substrate to fabricate a Mo/p-CZTS/n-Si/Al heterostructured solar cell. 

**Figure 7:**
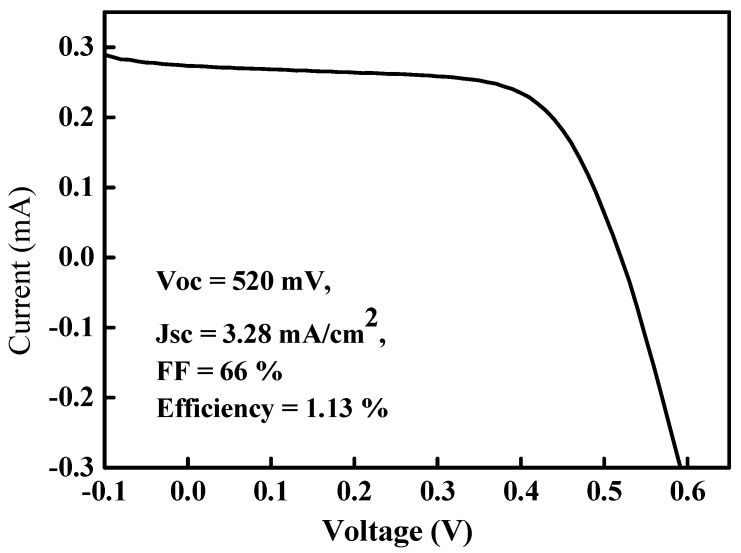
Current-voltage (*I*–*V*) characteristics of a Mo/CZTS/Si/Al heterostructured solar cell under (air mass) AM 1.5 G.

Figure 7 shows the (current-voltage) *I*–*V* characteristics of this Mo/CZTS/Si/Al solar cell under (air mass) AM 1.5 G. The open circuit voltage, short current density, fill factor, and efficiency were 520 mV, 3.28 mA/cm^2^, 66%, and 1.13%, respectively. The Mo/CZTS/Si/Al heterostructured solar cell prepared in this work was simple and the characteristics of the CZTS thin film used as the absorber in this solar cell should be investigated as soon as possible. Compared with the techniques of preparing CZTS thin films reported by other authors, our CZTS thin film preparation method of using spin-coating exhibits superior performance for photovoltaic solar cell applications.

## 3. Experimental Details

### 3.1. Preparation of CZTS Thin Film 

Because the solubility levels of CuCl_2_, ZnCl_2_, SnCl_2_, and thiourea powder in deionized (DI) water differ, these powders were dissolved in DI water individually to form aqueous precursor solutions with varied molar concentrations. If the aqueous precursor solutions do not contain toxic organic materials, such as MEA, spin-coating them uniformly onto glass substrates can be difficult. To spread precursor solutions uniformly without using toxic materials, the surfaces of the glass substrates were modified by using plasma cleaning to generate hydrophilic surfaces. To increase the S content of the CZTS thin film and prevent the mixed aqueous solution from becoming turbid, two separate spin-coating steps were applied to deposit CZTS thin films. For the first step, CuCl_2_, ZnCl_2_, and SnCl_2_ aqueous precursor solutions were mixed and stirred uniformly to form a metallic aqueous solution that was spin-coated onto soda lime glass substrates. The glass substrates, coated with uniformly distributed metallic aqueous solution, were treated using thermal processes, namely baking on a hot plate at 100 °C for 3 min to dewater and annealing in a furnace at 350 °C for 5 min to tailor the crystallinity and morphology of the CZT layer. In the second step, aqueous thiourea solution was spun onto the CZT layers, and then the samples were treated by being baked on a hot plate for 3 min at 100 °C. Then, thermal annealed in a furnace for 180 min at 350 °C to form CZTS thin films. The surface morphologies of the CZTS thin films were characterized using FE-SEM and XRD patterns by using a Bruker D8 advanced diffractometer (XRD: Bruker AXS-DS Discov, Taichung Taiwan) equipped with a CuKá (λ = 0.154 nm). The atomic contents of Cu, Zn, Sn, and S in these CZTS thin films were measured using EDX with the same diffractometer. The absorption of CZTS thin films in the visual range was measured using a UV–Vis–NIR spectrophotometer (UVD-350, Yunlin Taiwan). The electrical properties of CZTS thin films, including carrier concentration, carrier mobility, and resistivity, were measured using Hall measurements (HMS-5000) at room temperature.

### 3.2. Fabrication of the Mo/CZTS/n-Si/Al Heterostructured Solar Cell

The preparation of the Mo/p-CZTS/n-Si/Al heterostructured solar cell can be described as follows. First, aluminum (Al) metal was deposited on the back of an n–Si substrate using a thermal evaporation process and then a thermal annealing treatment was applied to the sample to form an ohmic contact between Si and Al. Second, the optimal CZTS absorber layer was deposited on the surface of the n-Si substrate through spin-coating, followed by a thermal annealing procedure. Third, the Mo metal was sputtered onto the CZTS absorber contact region to serve as ohmic contact metal. The sample was then annealed in N_2_ atmosphere for 5 min at 400 °C.

## 4. Conclusions

In this study, we spin-coated CZTS thin films onto glass substrates and annealed them to improve the crystallinity and morphology of the thin films. The amounts of Cu, Zn, Sn, and S in these CZTS thin films depend on the CuCl_2_, ZnCl_2_, SnCl_2_, and SC(NH_2_)_2_ concentrations of the precursor solutions. The surface morphology and optical band gap properties of these CZTS thin films relate to the Cu content and the electrical properties of these CZTS thin films depend on the annealing temperature and the Cu/(Zn + Sn) and Zn/Sn ratios. All of the CZTS thin films exhibited a polycrystalline kesterite crystal structure with three major broad peaks along the (112), (220), and (312) planes in XRD patterns. The optimal CZTS thin film can be deposited on a glass and Si substrate with proportions of [CuCl_2_] = 0.14 M, [ZnCl_2_] = 0.07 M, [SnCl_2_] = 0.07 M, and [SC(NH_2_)_2_] = 0.8 M and an annealing temperature of 350 °C. Finally, an optimal CZTS thin film was deposited on an n-type silicon substrate to produce a Mo/p-CZTS/n-Si/Al heterostructured solar cell with a conversion efficiency of 1.13% and with *V*_oc_ = 520 mV, *J*_sc_ = 3.28 mA/cm^2^, and fill-factor (FF) = 66%.

## Figures and Tables

**Table 1:** The S/(Cu + Zn + Sn) atomic content ratio and electrical characteristics of copper-zinc-tin-sulfur (CZTS) thin films with different thiourea concentration.

Thiourea Concentration (M)	S/(Cu + Zn + Sn)	Conduction Type	Carrier Concentration (cm^−3^)	Mobility (cm^2^/Vs)	Resistivity (Ùcm)
0.6	1.04	p/n	9.13 × 10^17^	0.34	20.7
0.7	1.07	p/n	1.32 × 10^18^	0.22	19.2
0.8	1.09	p	1.07 × 10^19^	0.49	1.19
0.9	1.19	p	8.87 × 10^18^	0.55	1.23

**Table 2:** Temperature dependence of conduction type, carrier concentration, mobility, and resistivity of the CZTS thin film measured using Hall measurements.

Annealing Temperature (°C)	Conduction Type	Carrier Concentration (cm^−3^)	Mobility (cm^2^/Vs)	Resistivity (Ùcm)
300	p/n	2.02 × 10^14^	126	245
350	p	1.07 × 10^19^	0.49	1.19
400	p/n	2.80 × 10^15^	7.95	280
450	p/n	1.24 × 10^16^	0.094	5310
